# Knowledge of HIV status prior to a community HIV counseling and testing intervention in a rural district of south Africa: results of a community based survey

**DOI:** 10.1186/1471-2334-12-73

**Published:** 2012-03-29

**Authors:** Hanani Tabana, Tanya Doherty, Sonja Swanevelder, Carl Lombard, Debra Jackson, Wanga Zembe, Reshma Naik

**Affiliations:** 1 Medical Research Council, Francie van Zyl Drive, Parrow, Cape Town, South Africa; 2 University of the Western Cape, Modderdam Road, Bellville, Cape Town, South Africa; 3 Division of Global Health (IHCAR), Department of Public Health Sciences, Karolinska Institutet, Nobels vag 9 5-171 77, Stockholm, Sweden; 4 School of Public Health, Boston University, 715 Albany Street, Boston 02118, MA, USA

## Abstract

**Background:**

The low uptake of facility-based HIV counseling and testing (HCT) in South Africa, particularly amongst men and youth has hindered attempts to increase access to effective treatment and prevention strategies. Many barriers to HIV testing have been described including long waiting times, transport to reach facilities, fear of lack of confidentiality and health systems factors such as stock outs of HIV test kits. The aim of this study was to undertake a community survey to determine rates of HCT in a rural area in order to plan a community intervention.

**Methods:**

A community-based survey was undertaken in 16 communities in Sisonke district, KwaZulu-Natal between September and November 2008. A total of 5821 individuals participated in the survey of which 66% were females. Gender specific mixed effects logistic regression models were used to describe differences in socio-economic characteristics, and their association with HIV testing histories.

**Results:**

Overall 1833 (32%) individuals in this rural area knew their HIV status. Prior testing was higher amongst women (39%) than amongst men (17%). Older men (> 24 years) were more likely to report having tested for HIV previously, with the highest likelihood (adjusted OR = 4.02; 95% CI: 2.71-5.99) among men in age group, 35-49 years. For women, age group 25-34 years had the highest likelihood of having been previously tested (adjusted OR = 1.30; 95% CI: 1.05-1.66). Being currently pregnant (adjusted OR 3.31; 95% CI: 2.29 - 4.78) or having a child under five (adjusted OR 7.00; 95% CI: 5.84 - 8.39) were also associated with prior HIV testing amongst women.

**Conclusions:**

Overall, knowledge of HIV status in this rural sub-district is low. The relatively higher uptake of HIV testing among women is encouraging as it shows that PMTCT services are well functioning. However, these data suggest that there is an urgent need for scaling up HIV testing services in rural communities specifically targeting men and youth.

## Background

HIV counseling and testing (HCT) has become an integral part of HIV prevention in sub-Saharan Africa [1] and an entry point to care, treatment and support for people living with HIV/AIDS. The low uptake of facility-based HCT in South Africa, particularly amongst young adults, has hindered attempts to increase access to effective treatment and prevention strategies. Many barriers to HIV testing have been described including social and economic barriers, and health systems factors [2]. Several strategies to increase uptake of HCT among sub-Saharan populations have been recommended [2] such as; mobile or community HCT, home-based testing, provider initiated HCT and routine (or opt-out) testing as part of medical care. These strategies have had varying degrees of success and their impact on the poorest rural populations may be limited. Enrollment of infected individuals into care therefore continues to be hampered by the low uptake of HCT [3] even in settings where HCT services are available [4-7].

South Africa currently has an overall national HIV prevalence of 17.8%, while the antenatal clinic (ANC) prevalence in women aged 15-49 years was 30.2% in 2010 [8]. These high HIV prevalence rates warrant interventions to increase the optimal use of HCT services. In an effort to address the high HIV prevalence, the South African government launched a national HCT campaign in 2010, targeting 15 million South Africans to test for HIV by June 2011 [9].

In many settings, HIV test uptake has been correlated with factors such as gender, education levels, geographical location, and number of sexual partners, amongst others [7,10]. A review on HCT uptake in sub-Saharan African countries using country level demographic and health survey (DHS) data reported that, in 26 countries in sub-Saharan Africa, 10% of women with no schooling reported having tested but throughout primary, secondary and higher education categories, testing uptake increased from 10% to 15%, 23% and 44% respectively. The review also reported that the findings based on the DHS wealth index showed that there was an increase in the likelihood of having had an HIV test with percentages ranging from 10% (poorest quintile) to 25% (wealthiest quintile). Testers were wealthier, more educated and less likely to be married [10].

In another study by Helleringer et al. 2009, reporting ever having tested for HIV at a health facility was correlated with; older age, increased number of schooling years, and having had multiple sexual partners over 3 years prior to the survey. During this study, when respondents were asked, they indicated distance to testing centre, lack of confidentiality in health facilities, and fear of diagnosis as reasons for not accessing services [3].

In South Africa, Venkatesh et al. 2011 [11] reported findings on characteristics of HIV 'testers' in a cross-sectional survey involving 1539 men and 1877 women in an urban township. Sixty eight percent of women reported prior testing compared to 29% of men. In this study, men and women who had heard about ARV therapy were more likely to report HIV testing, and repeated testing. Men who had more than 12 years of education and who were of high economic status, and women who were married and were of low economic status, and who had children under their care were more likely to have tested previously.

There is limited literature on HCT uptake in rural populations in South Africa. This paper reports findings from a baseline survey to ascertain prior HIV testing and predictors of knowledge of HIV status amongst inhabitants of the rural UMzimkhulu sub-district of KwaZulu-Natal, the province with the highest HIV prevalence in South Africa, prior to the implementation of a home-based HCT intervention trial.

## Methods

### Study design

A community-based survey was undertaken in 16 communities in Sisonke district, KwaZulu-Natal between September and November 2008. The community survey was implemented in one sub-district, UMzimkhulu within Sisonke, one of the poorest rural areas in South Africa, where 77% of households live below the poverty line [12]. Maps showing population numbers based on the Statistics South Africa (Stats SA) census were used to demarcate clusters with approximately 150 households in each. The purpose of the survey was to collect baseline information on knowledge of HIV status prior to implementation of a home based HCT intervention trial known as the Good Start study (ISRCTN31271935).

### Data collection

One data collector was assigned to each of the 16 clusters. She visited all households in a systematic manner and enumerated them using a household listing form obtained from Statistics South Africa. Upon reaching a household, all members of the household aged 18 years and older were asked for verbal consent to be interviewed. Data was collected using interviewer administered questionnaires uploaded on cell phones and transmitted via GPRS into a central database.

Interviews took place in a private place in the participant's home. The interviews were administered in Zulu or Xhosa, the local languages in the study area. Questions in the interviews included demographic characteristics of study participants (age, gender, and education), socio-economic status indicators (infrastructure and individual assets owned) and previous HIV testing history. Ethical approval for the survey was obtained from the University of the Western Cape research ethics committee.

### Statistical analyses

The primary outcome was a binary variable, "previous HIV testing" and "No previous HIV testing". All data analysis was conducted using STATA version 10 software (StataCorp LP, USA). Univariate and multivariate logistic regression was used to investigate factors associated with previous HIV testing. Gender specific models were fitted and included socio-demographic characteristics (age, educational attainment, socio-economic status) infant deaths in households, current pregnancy, household births, and children under 5 in the household. The dependency structure of the data relating to clusters and households was taken into account by including random effects in the models. All variables that were statistically significant in univariate analyses were included in multivariate analysis. In case of collinearity, one of the variables was dropped from the model. Prior to logistic regression model building, we conducted a TREE regression analysis which indicated major interactions between 'gender' and some socio-demographic variables. Based on these results and the fact that some variables were only applicable to women; we computed gender specific mixed effects logistic regression models and report odds ratios and 95% confidence intervals (CI). A post estimation test, "Wald test" for simple and composite linear hypotheses about the parameters of a fitted model was computed to compare levels of multilevel variables.

## Results

### Characteristics of study participants

Three thousand and forty nine households were approached and 86% of these were included in the survey. Very few households refused to take part (< 1%) and the most common reason (93%) for not including a household in the survey was that the owners were living and working in an urban area and the house was vacant. Out of the 2651 included households, a total of 5821 individuals were interviewed of which 3863 (66%) were females. Sixty-nine percent of the sample used rivers or a communal tap for drinking water and 63% used wood for cooking fuel. The mean age of men was 42 years (range 18-100 years) and the mean age of women was 39 years (range 18-95 years). Seventy four percent of men and women had some primary schooling or had completed primary school.

### History of HIV testing

**Figure 1:**
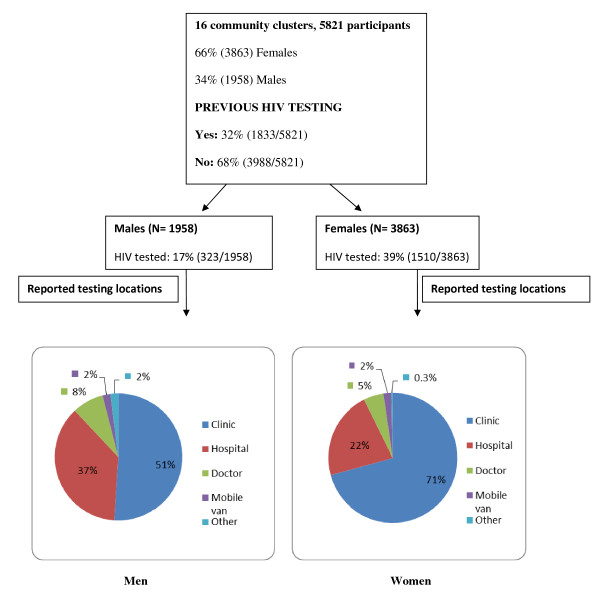
**Distribution of participants (by gender, report of previous HIV testing and location of testing) enrolled in the study**.

Of the total sample, 1833 (32%) people reported having tested for HIV previously. Of those who had tested previously, 82% were women. Among men, only 17% (323/1958) had previously tested for HIV, while for women this rate was 39% (1510/3863). Encouragingly 75% of women who reported being pregnant at the time of the survey had tested for HIV. The most common place for people to test (77%) was clinics and hospitals. Figure 1 shows the distribution of location where HIV testing was done by gender.

### Predictors of prior HIV testing

Tables 1 and 2 show univariate and multivariate analysis of socio-demographic characteristics associated with previous HIV testing among men and women respectively. Predictors of testing varied by gender and therefore are presented separately. In multivariate analysis, older men (> 24 years) were more likely to report having tested for HIV previously (compared with 18-24 years), with the highest likelihood (adjusted OR 4.02; 95% CI: 2.71-5.99) among men in age group 35-49 years (Table 1). For women, age group 25-34 years (compared with 18-24 years) had the highest likelihood of having been previously tested (adjusted OR 1.3; 95% CI: 1.05-1.66). Women older than 50 years were less likely (adjusted OR 0.39; 95% CI: 0.31- 0.51) to report ever testing for HIV previously (Table 2). The post estimation test indicated that there were significant (P < 0.05) differences between successive age groups 25-34 and 35-49 years among women, and also between age groups 25-34 and 35-49 years among men.

**Table 1:** Univariate and multivariate analysis of socio-demographic characteristics associated with previous HIV testing among MEN in rural Sisonke district, South Africa (n = 1958)

Characteristic	Proportion reporting		
	**previous HIV testing**	**Crude OR**	**Adjusted OR**

	**Yes, n/N (%)**	**OR (95% CI)**	**OR (95% CI)**

**Age group, yrs**			

18-24	42/570 (7.4)	1.00	

25-34	81/370 (21.9)	**3.52 (2.59-4.79)**	**3.44 (2.30-5.18)**

35-49	102/430 (23.7)	**3.91 (2.94-5.20)**	**4.02 (2.71-5.99)**

50+	98/588 (16.7)	**2.51 (1.73 -3.65)**	**2.62 (1.76-3.90)**

**Education Level**			

None	21/135 (15.6)	1.00	

Primary school	234/1442 (16.2)	**1.05 (0.73-1.51)**	**1.22 (0.74-2.03)**

Completed High school	48/289 (16.6)	**1.08 (0.61-1.91)**	**1.28 (0.69-2.36)**

Tertiary	20/92 (21.7)	**1.51 (0.86-2.65)**	**1.17 (0.58-2.39)**

**Drinking water source**			

All other sources	253/1647 (15.4)	1.00	

Piped (inside house and yard)	70/311 (22.5)	**1.60 (1.09-2.34)**	**1.66 (1.14-2.4)**

**Cooking fuel**			

Wood	174/1195 (14.6)	1.00	

Paraffin/Kerosene/Gas	41/215 (19.1)	**1.38 (0.69-2.75)**	**1.18 (0.77-1.78)**

Electricity	108/548 (19.7)	**1.44 (0.94-2.21)**	**1.42 (1.04-1.93)**

**Infant deaths in past year**			

No	279/1740 (16.0)	1.00	

Yes	44/218 (20.2)	**1.32 (0.92-1.91)**	**1.25 (0.86-1.80)**

**Infant births in past year**			

**No**	128/970 (13.2)	1.00	

**Yes**	57/340 (16.8)	**1.32 (0.89-1.96)**	

**No. of people living together**			

< = 2	134/738 (18.2)	1.00	

> 2	189/1220 (15.5)	**0.83 (0.62-1.09)**	

**Household wall material**			

Other	10/69 (14.5)	1.00	

Mud/wood/grass	107/600 (17.8)	**1.28 (0.81-2.03)**	

Brick/cement/corrugated iron	206/1289 (16.0)	**1.12 (0.63-1.98)**	

**Refrigerator**			

No	181/1097 (16.5)	1.00	

Yes	142/861 (16.5)	**0.99 (0.82-1.22)**	

**Radio**			

No	80/488 (16.4)	1.00	

Yes	243/1470 (16.5)	**1.01 (0.77-1.33)**	

**Television**			

No	152/853 (17.8)	1.00	

Yes	171/1105 (15.5)	**0.84 (0.63-1.13)**	

**Cell phone**			

No	61/374 (16.3)	1.00	

Yes	262/1584 (16.5)	**1.02 (0.68-1.51)**	

**Stove**			

No	80/456 (17.5)	1.00	

Yes	243/1584 (16.2)	**0.91 (0.66-1.24)**	

**Cupboard**			

No	97/527 (18.4)	1.00	

Yes	226/1431 (15.8)	**0.83 (0.61-1.14)**	

**Table 2:** Univariate and multivariate analysis of socio-demographic characteristics associated with previous HIV testing among WOMEN in rural Sisonke district, South Africa

Characteristic	Proportion reporting		
	**previous HIV testing**	**Crude OR**	**Adjusted OR**

	**Yes, n/N (%)**	**OR (95% CI)**	**OR (95% CI)**

**Age group, yrs**			

18-24	488/905 (54.0)	1.00	

25-34	473/735 (64.4)	**1.54 (1.27-1.88)**	**1.32 (1.05-1.66)**

35-49	368/898 (41.0)	**0.59 (0.47-0.75)**	**1.00 (0.80-1.25)**

50+	181/1325 (13.7)	**0.14 (0.10-0.18)**	**0.39 (0.31-0.51)**

None	28/233 (12.0)	1.00	

Primary school	1082/2846 (38.0)	**4.49 (2.41-8.37)**	**1.82 (1.16-2.87)**

Completed High school	319/608 (52.5)	**8.08 (4.57-14.29)**	**2.03 (1.23- 3.36)**

Tertiary	81/174 (46.6)	**6.38 (3.62-11.24)**	**2.99 (1.68-5.33)**

All other sources	1334/3367 (39.6)	1.00	

Piped (inside house and yard)	176/496 (35.5)	**0.83 (0.69-1.02)**	**0.88 (0.65-1.18)**

< = 2	784/1943 (40.4)		

> 2	726/1920 (38.0)		

Wood	974/2490 (39.1)	1.00	

Paraffin/Kerosene/Gas	149/400 (37.3)	**0.92 (0.69-1.23)**	**0.96 (0.72-1.28)**

Electricity	387/973 (39.8)	**1.03 (0.78-1.36)**	**1.05 (0.85-1.31)**

**No**			

**Yes**	1317/3394 (38.8)		

193/469 (41.2)	1.00		

**0.91 (0.68-1.21)**	**1.14 (0.89-1.44)**		

**Child Under 5**			

**No**	588/2651 (22.2)	1.00	

**Yes**	922/1212 (76.1)	**11.15 (7.23-17.20)**	**7.00 (5.84-8.39)**

**Currently pregnant**			

**No**	1358/3660 (37.1)	**1.00**	

**Yes**	152/203 (74.9)	**5.05 (2.91-8.76)**	**3.31 (2.29-4.78)**

**Infant births in past year**			

**No**	575/1856 (30.9)	1.00	

**Yes**	403/757 (53.2)	**2.54 (2.01-3.19)**	

No	942/2303 (40.9)	1.00	

Yes			

**Radio**	568/1560 (36.4)	**0.83 (0.69-0.99)**	

No	423/967 (43.7)	1.00	

Yes	1087/2896 (37.5)	**0.77 (0.65-0.92)**	

Yes	772/2039 (37.9)	**0.89 (0.74-1.08)**	

**Cell phone**			

Yes	1284/3149 (40.8)	**1.49 (1.22-1.81)**	

**Stove**			

Yes	1155/2870 (38.9)	**0.96 (0.81-1.16)**	

**Cupboard**			

Yes	1093/2846 (38.4)	**0.89 (0.73-1.11)**	

Education was not associated with testing in men but was associated with testing in women with education levels, "completed high school" and "completed tertiary" having adjusted OR 2.03; 95% CI: 1.15-2.87 and adjusted OR 2.99; 95% CI: 1.68-5.33 respectively compared to no education.

Socio-economic factors were associated with testing history for men and women. Among men using electricity as primary cooking fuel compared to those using wood (adjusted OR 1.42; 95% CI: 1.04-1.93) was positively associated with testing as well as having piped water inside the house or yard compared with all other sources of water (adjusted OR 1.66; 95% CI: 1.14-2.40) (Table 1). In women neither drinking water source nor cooking fuel was predictive of prior HIV testing (Table 2). However, women with a cell phone were more likely to have tested (OR 1.49; 95% CI: 1.22-1.81).

For women, current pregnancy (adjusted OR 3.31; 95% CI: 2.29-4.78) and having a child less than 5 years of age (adjusted OR 7.00; 95% CI: 5.84- 8.39) were strongly associated with previous HIV testing. Giving birth in the past year was also predictive of prior HIV testing (adjusted OR 2.54; 95% CI: 2.01-3.19).

## Discussion

This large community based survey in rural South Africa has found that only 32% of adults had ever been tested for HIV previously. This is concerning, especially in the province with the highest HIV prevalence in South Africa [8]. The most recent National HIV, behavior and health survey in South Africa found that in KwaZulu-Natal province, only 24% of adults in the age group 15-49 years had tested for HIV in the past year [13]. We found an even lower rate of HIV testing (17%) amongst men which could be attributable to the fact that in this rural area with limited access to non clinical HIV testing services, men are unlikely to voluntarily test for HIV unless they are ill and attend health care services. Another possible explanation could be due to the common phenomenon of 'proxy' testing among men which has been reported in South Africa, where men entrust testing to their partners assuming that their status would be the same as that of their partners [14].

For many years, primary health clinics and hospitals have provided the only access to HCT in South Africa and other high HIV prevalence settings hence the majority of participants who reported prior HIV testing had tested at a clinic or hospital. Given the low rate of testing under these circumstances the shift to non-clinical venues for HCT [2] is critical to increase population level coverage of HIV testing.

Our findings show that the most significant predictor of previous HIV testing was age group. Among men, those 35-49 years, and among women, those 25-34 years were more likely to test compared to those 18-24 years. Women in the age group 25-34 years are mostly in the child bearing age, hence it is expected that these women may have been tested in the antenatal clinic. Women of reproductive age are more likely to come into frequent contact with the health system where they are offered HCT services. However, among women, being older (age groups 50+ years compared to age group 18-24 years) was associated with a relatively lower likelihood to test. These findings of less testing in older age groups is consistent with the literature [15-17] and needs further exploration as high HIV prevalence rates have been previously reported in this age group [16,17]. A possible explanation for less testing amongst older women could be that older people are often in stable relationships and have a lower HIV risk perception. Furthermore, as compared with the present day, HIV testing of pregnant women in South Africa was not routine prior to 2001 when these older women would have been in their child bearing years.

Previous surveys in South Africa have reported risky sexual behaviours among young people, between the ages 15-24 years [13,18,19]. Therefore the finding of low levels of testing amongst young men is concerning and strategies specifically targeted at this group are urgently needed.

We found inconsistencies across gender for socio-demographic factors associated with HIV testing such as education, and infrastructure. Higher education was associated with testing in women but not in men. Higher education level is consistently seen in the literature to be associated with HIV testing [7,10,11,20] though there have been conflicting results in other African settings, where having higher education was associated with a lower likelihood to undergo HCT [21]. Furthermore, we found men who had electricity and piped water to have a greater likelihood to have tested but these factors were not associated with testing in women. We used factors like electricity and piped water as a proxy for socio-economic status, and these findings may suggest that being of higher socio-economic status, better enabled men to test, whereas most women might have had to test at some point in their lifetimes during pregnancy regardless of socio-economic standing.

It is quite encouraging that current pregnancy and having a child under 5 was highly predictive of prior testing among women and this indicates that prevention of mother to child transmission of HIV (PMTCT) services are working well. HIV testing amongst antenatal clients in South Africa has increased from 49% in 2004 to an average of 100% across all districts in the country in 2010/2011 [22]. However, the PMTCT programme is clearly not involving men, as a birth in the last year was not associated with HIV testing in men. Future HIV testing strategies should include scale up of couple counseling and testing in antenatal care settings as well as home-based couples testing to reach more men, especially those in the reproductive age. Furthermore in rural female dominated settings similar to the current study setting, where men largely work in the cities and are not at home for most of the year, interventions need to operate over holidays to reach these men.

The greatest strength of this study is that it was a large community wide survey with a high participation rate in a hard to reach rural area. However, there are some limitations to mention. We did not ask the participants why they tested or did not test and are thus unable to understand the underlying motivations to test or avoid testing in this specific population. It would also have been valuable to ask participants how recently they had tested since this would have indicated whether individuals are testing regularly according to the national guidelines. Since people may have tested several years prior to the survey, recall bias regarding place of testing is possible. Further research is needed, possibly qualitative, to explore motivations for testing or not testing in rural populations.

## Conclusion

This study has shown low knowledge of HIV status, especially amongst men, in a poor rural area of South Africa. There is an urgent need to scale up HIV testing services in rural areas using community based approaches specifically targeting young men in order to increase access to HIV care and treatment and to promote safer sexual behavior amongst this high risk group.

## Competing interests

The authors declare that they have no competing interests.

## Authors' contributions

HT, TD, DJ, WZ and RN planned and wrote the paper. SS and CL were study statisticians. DJ, TD and HT contributed to manuscript design and content. HT, WZ, and RN had particular responsibility for study implementation. HT, RN and WZ were active with data management and analytic content. TD and DJ were principal investigators and planned the study design, oversaw implementation and worked on analytic content. All authors read and contributed towards the final draft. All authors read and approved the final manuscript.

## Pre-publication history

The pre-publication history for this paper can be accessed here:

http://www.biomedcentral.com/1471-2334/12/73/prepub
